# Microspatial distribution of trace elements in feline dental hard tissues: early life exposure to essential and toxic elements

**DOI:** 10.3389/fvets.2023.1204210

**Published:** 2023-06-27

**Authors:** Alexandra L. Wright, Nadine Fiani, Santiago Peralta, Manish Arora, Christine Austin

**Affiliations:** ^1^Department of Clinical Sciences, College of Veterinary Medicine, Cornell University, Ithaca, NY, United States; ^2^Environmental Medicine and Public Health, Icahn School of Medicine at Mount Sinai, New York, NY, United States; ^3^Icahn School of Medicine at Mount Sinai, Institute for Exposomic Research, New York, NY, United States

**Keywords:** feline, trace elements, dental, exposure, toxin, mass spectromotry

## Abstract

**Introduction:**

Trace elements play a key role in dental tissue development, as dental hard tissues accumulate both essential and toxic trace elements during mineralization. Characterization of the spatial accumulation pattern of trace elements may provide insight into exposure to toxic elements over time and to the nature of disease processes affecting the hard dental tissues. Here, we present the first report of the use of laser ablation-inductively coupled plasma-mass spectrometry (LA-ICP-MS) to map the microspatial distribution of multiple trace elements, essential and toxic, across feline dental hard tissues.

**Methods:**

Eleven teeth were extracted from 8 cats. Nine teeth were from 7 cats diagnosed with idiopathic tooth resorption on intraoral radiographs prior to extraction. Two teeth were included from a cadaver that had no signs of tooth resorption on intraoral radiographs. The normal dental tissue was analyzed from each sample using LA-ICP-MS to map the microspatial distribution of essential and toxic trace elements across feline enamel, dentin, and cementum.

**Results:**

Results showed a higher accumulation of barium and strontium in coronal dentin as compared to root dentin. The timing of the accumulation mirrors nursing timelines seen in teeth from human and non-human primates, consistent with barium and strontium being sourced from maternal milk. Results also showed a higher uptake of lead in the coronal dentin, suggesting this lead exposure was likely passed from mother to offspring.

**Discussion:**

This work characterizes a baseline for elemental distribution in feline teeth linked to early life exposure to toxic elements such as lead and provides a framework for future studies investigating long-term environmental exposures to trace elements, essential and toxic, and their involvement in feline systemic and dental diseases.

## Introduction

Dental disease can be debilitating in humans and domestic animals alike, and interruptions in tooth development can predispose individuals to caries, tooth loss, oral pain, and other morbidities (1–3). Trace elements are known to play an important role in mammalian tooth development as they are incorporated into dental hard tissues during mineralization (4–7). The formation and mineralization of the hard dental tissues follow a specific chronologic order during odontogenesis and then post-eruption. Crown formation occurs first during tooth development as ameloblasts facilitate calcium and magnesium ion transport for hydroxyapatite crystal formation in enamel and dentin (4, 6, 7). Dentin formation continues to the roots, and cementum forms the peripheral layer of the root and undergoes biomineralization (4, 8). Dentin accumulates trace elements over time post-eruption as secondary dentin is continually produced and mineralized while the tooth remains vital (4, 7). Several trace elements, such as strontium and barium, have been shown to replace calcium in the hydroxyapatite crystal of mineralized dental tissues (7), thus affecting the structural integrity of the teeth and possibly predisposing to dental disease.

In humans, it has been shown that metabolic disorders leading to disruption in the optimal balance of essential elements can cause dental pathologies such as caries and periodontal disease (9–15). Elements are often incorporated through interactions involving shared transport pathways. For example, lead and zinc ions are known to compete with calcium and phosphorus ions, thus potentially creating an offset in normal levels of these essential elements in teeth when lead or zinc is in excess (7, 12, 16). This may explain why exposure to metals such as lead and cadmium has been linked to a higher prevalence in pediatric dental caries (11). Additionally, another study showed higher concentrations of copper and zinc in diseased dentin of carious lesions when compared with normal dentin in the non-diseased portion of the tooth (17). These findings in humans suggest that examining the deposition patterns of trace elements in teeth can provide insight into their potential role in dental disease in other species as well.

There has been a growing interest in investigating the exposure of companion animals to trace elements in their diet (18–20). Most studies have focused on quantifying toxic elements, such as lead, arsenic, and cadmium, in different commercial pet foods, revealing the levels to be below the regulatory guidelines. However, low levels of food still have the potential to cause chronic or cumulative exposure, generating a recent call for further research to assess the potential long-term health impact of metal exposure from the diet for animals (20). Feline oral health has been a particular area of interest in veterinary medicine as cats have become more popular as companion animals (21), and their quality of life can be significantly affected by dental disease. One common ailment of the cat is tooth resorption, a painful, progressive disease of the dental hard tissues, affecting an estimated 20–67% of the general feline population (22, 23). Cats can present with halitosis, dysphagia, ptyalism, anorexia, and weight loss, but often they do not show overt clinical signs. Multiple environmental and biological factors have been hypothesized to play a role including diet, geography (city vs. rural), oral pH, endocrine disease, viruses, vitamin D, and cytokines (24–27), but the primary etiopathogenesis of these lesions remains unknown. The high prevalence and morbidity these lesions inflict on this companion animal species make further investigation of the disease imperative to veterinary practitioners.

A first step in examining the potential role of trace elements in feline dental disorders is adapting available technologies designed to determine the uptake and spatial distribution of trace elements in dental hard tissues. Dental hard tissues are relatively stable over time, and to a large extent, the metals deposited in teeth during mineralization are retained. The known formation timing of the different dental tissue layers offers a record of exposure to environmental pollutants including heavy metals and other trace elements deposited during mineralization (4). Laser ablation-inductively coupled plasma-mass spectrometry (LA-ICP-MS) is a well-established technique used to map elements in teeth, as well as other biological tissues, at a micrometer spatial resolution (28–30). Thus, the distribution of trace elements along histologic layers in teeth has been applied mainly to rodent, human, primate, and most recently equine teeth (16, 31–35). This study aimed to establish the feasibility of the use of LA-ICP-MS to measure trace element levels in feline hard dental tissues (enamel, dentin, and cementum) and to characterize the microspatial distribution of tooth-seeking trace elements (lead, strontium, barium, and zinc) in feline teeth.

## Methods

### Study population and tooth extraction

For this study, we focused on the distribution of trace elements in the disease-free sections of hard dental tissues. Feline teeth were included in this study from one cadaver specimen that had been euthanized for non-dental-related reasons and feline patients that had undergone periodontal assessment and treatment at Cornell University Companion Animal Hospital (Table 1). All cadaver teeth were confirmed to be radiographically normal prior to extraction. The teeth were, then, extracted by a standard surgical technique, including sectioning of multi-rooted teeth at the furcation. Teeth from seven live patients that were diagnosed with idiopathic tooth resorption preoperatively with intraoral radiography were included in this study, following the treatment via extraction of the affected teeth. It was required that the samples used for analysis included the dentinoenamel junction (DEJ) and cementoenamel junction (CEJ). If the tooth could not be extracted with the CEJ intact, then it was excluded from the laboratory analysis. Teeth collected were extracted between January 2021 and September 2021 by dentistry and oral surgery residents in training under the supervision of board-certified veterinary dentistry specialists. Samples were packaged individually, labeled, and assigned a study enrollment number to keep patient signalment and medical history blind to individuals processing and analyzing all the teeth. The teeth were stored dry at ambient room temperature and away from any intense light to avoid heat prior to processing and analysis.

**Table 1:** Summary of teeth included in this study.

	**Cadaver vs. live patient**	**# of teeth collected**	**Tooth type**	**Diseased vs. non-diseased teeth**
1	Live patient	1	Mandibular first molar	Diseased
2	Live patient	2	Maxillary fourth premolar, mandibular canine	Diseased
3	Live patient	1	Mandibular first molar	Diseased
4	Live patient	1	Maxillary fourth premolar	Diseased
5	Cadaver	2	Two mandibular first molars	Non-diseased
6	Live patient	2	Maxillary fourth premolar, mandibular fourth premolar	Diseased
7	Live patient	1	Maxillary third premolar	Diseased
8	Live patient	1	Mandibular canine	Diseased

Multiple teeth included from the same animal were analyzed for reproducibility of trace element distribution in the dental hard tissues of that animal. Teeth labeled as “diseased” indicate a diagnosis of idiopathic tooth resorption of that tooth and subsequent extraction as treatment. “Non-diseased” cadaveric teeth had no signs of tooth resorption on the radiograph.

### Tooth preparation and laboratory analysis for trace elements

Trace metal-free water produced through reverse osmosis (MilliQ water) was used to clean the teeth and avoid external contamination during laboratory processing. Teeth were then sectioned along the labiolingual plane using a slow-speed rotary saw (IsoMet, Buehler) with a diamond-tipped blade. One half of the tooth was embedded in epoxy resin, and the cut surface was polished down to 1 μm roughness with diamond paste. An ESL NWR193 laser ablation system equipped with a Coherent ExciStar argon fluoride excimer laser emitting at 193 nm was used. The laser ablation unit was connected to an Agilent Technologies 8800 triple-quadrupole ICP-MS using Tygon^®^ tubing. Details of the analytical methods have been published previously (36).

**Figure 1:**
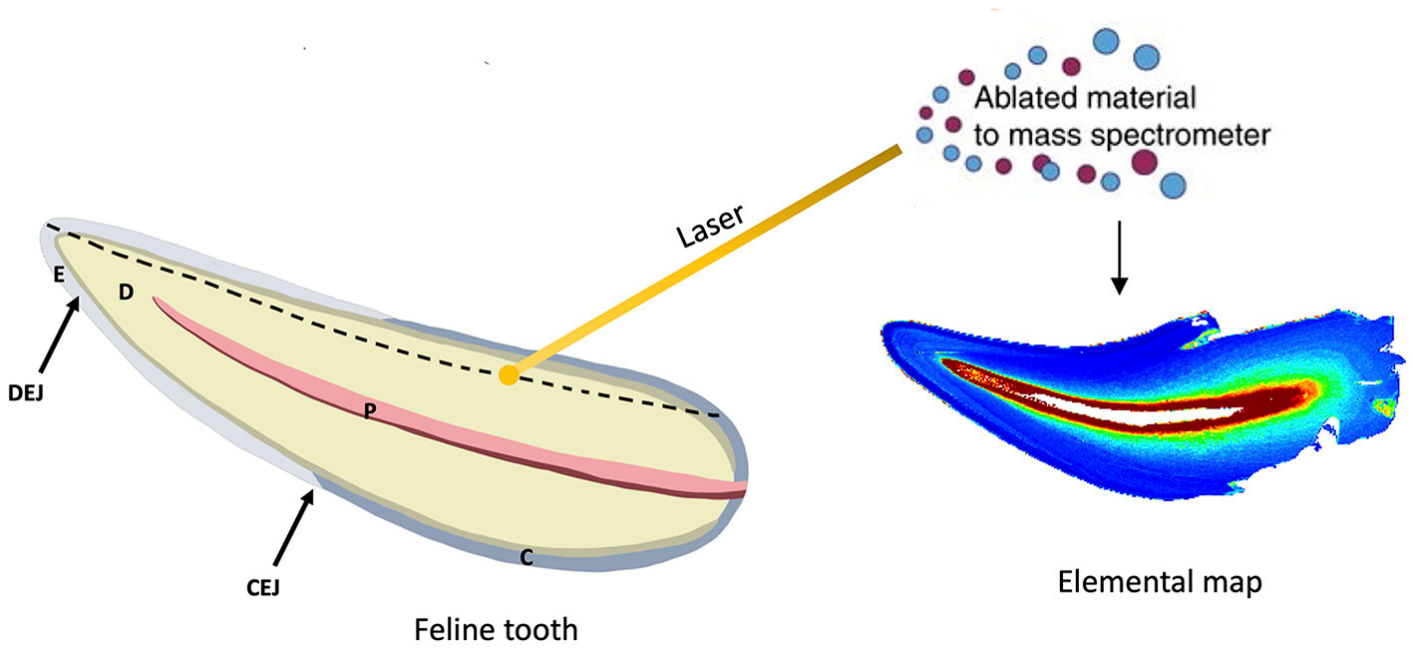
Overview of methods. Extracted feline teeth were sectioned, embedded in epoxy resin, and polished to a flat surface. Illustration of a sectioned feline tooth and its components depicted: enamel (E), dentin (D), pulp (P) cementum (C), dentinoenamel junction (DEJ), and cementoenamel junction (CEJ). LA-ICP-MS was used to generate microspatial elemental maps to determine the distribution of trace elements across non-diseased mineralized dental tissue. Relative concentrations of the denoted element are visualized via a color gradient (blue depicts lower concentrations, and red depicts higher concentrations). Laser sampling of the dental tissue was performed from the crown to the apex, depicted by the dotted line. Several adjacent linear scans combined produce the elemental map. Pulp is not mineralized and therefore presents as red on the elemental map as there is no metal:calcium ratio present.

For the LA-ICP-MS analysis, the entire cut surface was rastered, taking thousands of sampling points of linear scans adjacent to each other (Figure 1). Once these linear scans were combined, two-dimensional elemental maps were generated. Subsequently, using this information as a guide, single linear traces were taken for all teeth from the dentin horn of the tooth crown to the CEJ. The results of these linear traces are shown in Supplementary Figures S1, S2. The LA-ICP-MS analysis generates metal intensity data in.csv format, which was then converted into images by assigning a color relative to the metal ion intensity at each sampling point. The color gradient of low-to-high concentrations is provided in the figures. The details of this method and the custom R code that was used to achieve this have been detailed previously (37). A laser spot size of 35 μm, scan speed of 80 μm s^−1^, repetition rate of 60 Hz, laser power of 2.4 J cm^−1^, and ICP-MS total integration time of 0.4375 s were used for the linear traces. Notably, the laser spot size is smaller than the reported thickness of feline enamel (38). The surface was first pre-ablated with a laser spot size of 50 μm, scan speed of 100 μm s^−1^, repetition rate of 3 Hz, and laser power of 0.8 J cm^−1^ to remove any surface contamination. Metals were normalized to calcium to account for individual mineral density variation within and between samples, and metal:calcium ratios are reported. Two teeth from the same cadaver specimen were analyzed to establish the reproducibility of trace element measurements.

## Results

**Figure 2:**
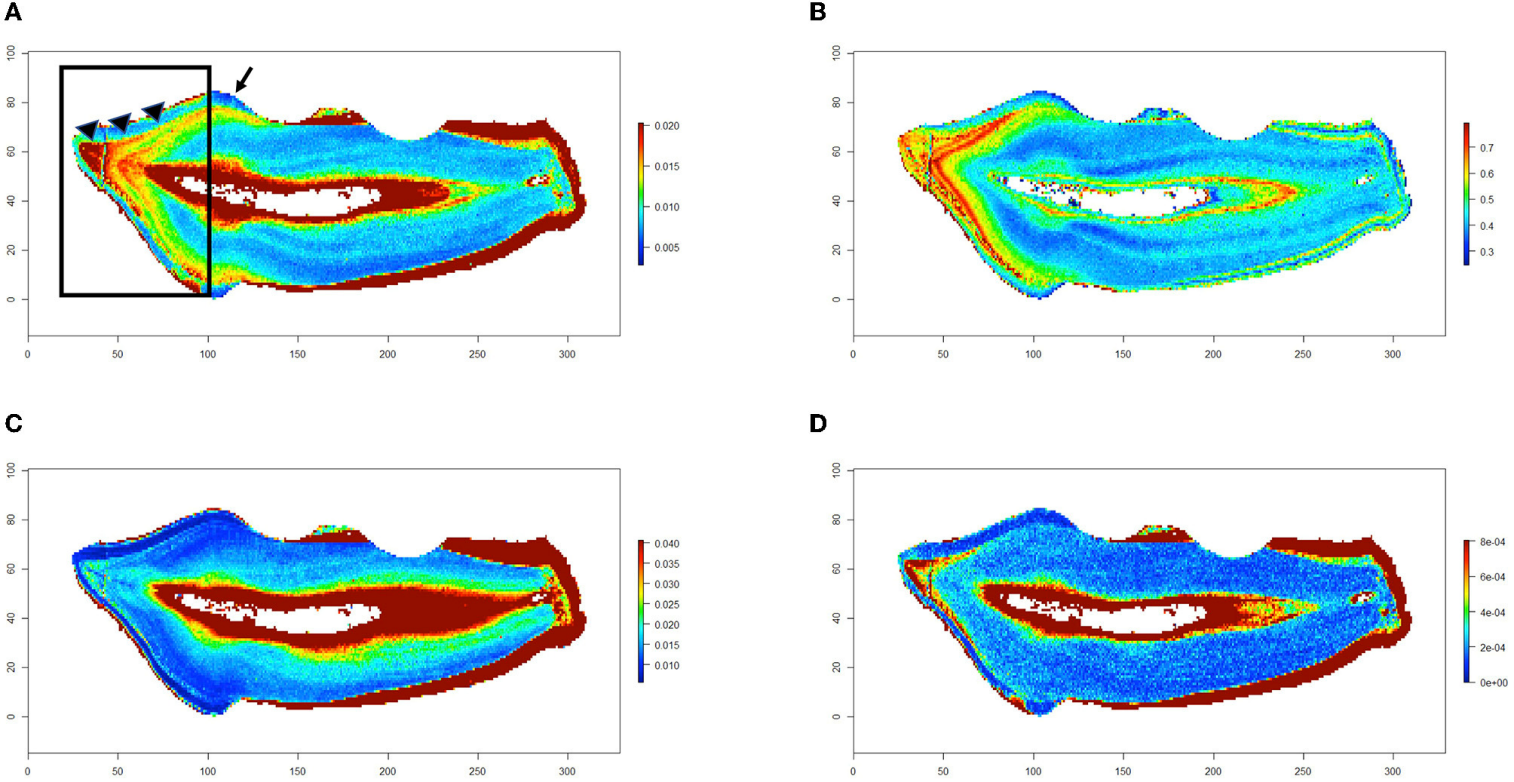
Trace elements in a non-diseased tooth. Trace elements in a healthy feline's mandibular first molar tooth. Maps for barium **(A)**, strontium **(B)**, zinc **(C)**, and lead **(D)** are shown. Relative concentrations of the denoted element are visualized via a color gradient on each map (blue depicts lower concentrations, and red depicts higher concentrations). Barium and strontium maps show higher concentration ratios in the coronal dentin (black box on barium map) starting at the DEJ (arrow heads) and tapering to a sharp decline in the concentration of that element at the CEJ (black arrow on barium map). This is consistent with uptake during nursing. Additionally, zinc and lead levels have a higher concentration in the coronal dentin at the same temporal band as the barium and strontium levels.

**Figure 3:**
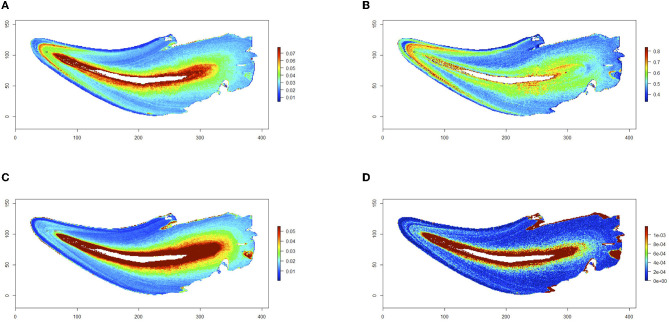
Trace elements in a diseased tooth. A mandibular canine tooth extracted from a cat due to tooth resorption lesions. Maps for barium **(A)**, strontium **(B)**, zinc **(C)**, and lead **(D)** are shown. Relative concentrations of the denoted element are visualized via a color gradient on each map (blue depicts lower concentrations, and red depicts higher concentrations). Non-diseased dental hard tissues were mapped. Higher uptake of barium and strontium is present in the coronal dentin from DEJ to CEJ when compared with root dentin, similar to that of the first molar tooth in Figure 2 and consistent with nursing timing. Banding patterns of higher concentrations of lead and zinc are noted at the same coronal dentin location as compared with the dentin of the root.

**Figure 4:**
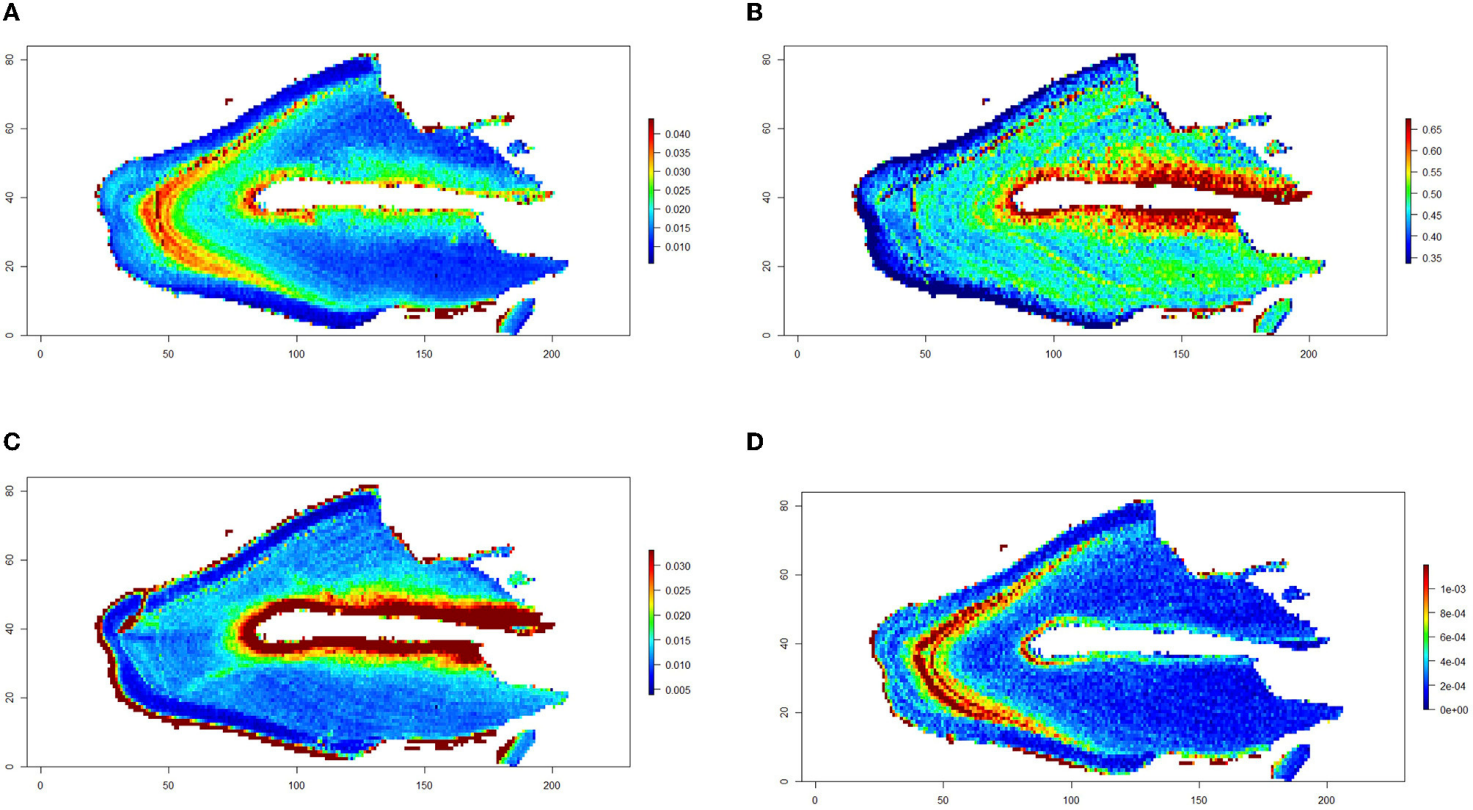
Trace elements in a diseased first molar tooth. A mandibular first molar tooth extracted from a cat due to tooth resorption lesions. Maps for barium **(A)**, strontium **(B)**, zinc **(C)**, and lead **(D)** are shown. Relative concentrations of the denoted element are visualized via a color gradient on each map (blue depicts lower concentrations, and red depicts higher concentrations). Non-diseased dental hard tissues were mapped. Higher uptake of barium, strontium, and lead is present in the coronal dentin from DEJ to CEJ.

The LA-ICP-MS analysis scans teeth in two dimensions to create maps of multiple trace elements across histologic layers in the same analytical scan. A color concentration scale is generated for each trace element mapped, indicating differences in the concentration of that element across the analyzed surface. The blue spectrum notes relatively lower concentrations, and the red spectrum notes higher concentrations of the denoted element of each map. The measurements at the periphery of the tooth are an artifact of the analysis, resulting in a high concentration reading from the sudden drop in calcium when no sampling dental tissue (calcium is used as an internal standard and is the denominator in the intensity measures). Eleven teeth were included in the study from a total of eight cats. Elemental maps were generated for three teeth, and we have provided each with image panels in Figures 2–4. Overall, feline teeth showed variations in the distribution of different elements in different parts of the tooth. We provide examples of four elements that are known to play key physiologic roles in dental development or are known toxicants. Specifically, we report on tooth-seeking elements (i.e., elements known to concentrate in teeth) such as lead, strontium, barium, and zinc. Distributions of trace elements varied markedly, showing areas of high and low uptake within the dental hard tissues of each tooth. The concentration of these elements is further evident from the intensity indices mapped visually, as banding patterns are noted when different histologic layers of dental tissue exhibit high- and low-intensity indices.

Trace element mapping was first performed in the first molar teeth collected from a cadaver specimen that was radiographically normal. Element distributions are visualized in Figure 2, showing a notable difference in barium and strontium levels in the coronal dentin. The banding pattern indicates that these higher concentrations followed the normal mineralization pattern of the tooth's development and maturation. The banding pattern shows a higher concentration of barium and strontium at the level of the DEJ of the crown and tapers to a loss of concentration at the level of the CEJ, where the crown meets the root. Barium and strontium are known to be in higher concentration during nursing, and the banding patterns present in these teeth show a temporal difference in barium and strontium levels. Additionally, in the cat's first molar tooth, there were higher levels of zinc and lead uptake in the earliest formed coronal dentin aligning with nursing timing as well (Figure 2). This banding pattern was evident in the coronal dentin of all teeth analyzed, confirming that the concentrations of trace elements correlate with the mineralization of that tooth's normal tissue. Though not always synchronous, discrete zones of high strontium, barium, and lead generally occurred within the earliest formed dentin and enamel of the crown, with lower levels in the root dentin, across all the teeth analyzed. The dentin immediately adjacent to the pulp and the cementum had the highest levels of zinc, barium, and lead.

The higher level of barium and strontium in the coronal dentin was noted in all tooth types (first molar vs. premolar vs. canine tooth), while the width of the banding pattern differed among them. The first molar teeth analyzed had the widest band of barium and strontium in the coronal dentin (Figure 2), indicating earlier mineralization at the time of barium and strontium exposure than the canine and premolar teeth. In the cat's canine tooth analyzed in Figure 3, a similar high concentration of barium and strontium is noted in the earliest formed coronal dentin. The uptake noted in this tooth is a thinner band, indicating a shorter period of time of uptake during mineralization in comparison with the first molar tooth. As with the molar tooth, the canine tooth also shows an increased uptake of lead and zinc in the earlier formed coronal dentin in tandem with the nursing timing pattern on the barium and strontium maps. Although a lower concentration of lead is noted in the coronal dentin of the canine tooth than that of the first molar tooth, it is still notably higher than the concentrations in the dentin of the root of the canine tooth.

Figure 4 represents the distribution of trace elements in the coronal dentin of a first molar that was extracted from a cat due to tooth resorptive lesions. The CEJ is intact, but the root could not be retained during resin-mounting processing. The non-diseased tissue present coronally showed distribution of barium and strontium, consistent with the pattern seen in the normal first molar tooth, where there is a higher uptake of these elements in the coronal dentin. Additionally, a high intensity of lead is noted in the same histologic location, indicating that this uptake was at the same time as barium and strontium, as noted in the other teeth. Due to the small size of feline teeth, the laser diameter used in this study could not measure the elemental distribution in the dentin immediately adjacent to resorptive lesions in a fine temporal resolution.

## Discussion

The use of LA-ICP-MS technology to study the microspatial distribution of trace elements in hard dental tissues provides unique insight into an individual's timeline of exposure due to the predictable mineralization process of teeth. Odontogenesis is a dynamic process involving the differentiation stages (bud, cap, and bell stages) of the tooth germ (6). It can also be largely divided into crown formation followed by root formation (39). As the tooth germ proliferates and matures, the secretory stage leads to the mineralization of the dental hard tissues (enamel, dentin, and cementum) (4, 6). Enamel formation ceases with the completion of crown formation, but dentin continues to form during root formation and internally as the tooth ages (39, 40). As teeth develop and mature, mineralized layers of dentin form present systemically into the crystalline structure while incorporating trace elements (4, 33). This preserves the timing and relative concentration of the element in the dentin-mineralized matrix, thus allowing our methods to map the levels of trace elements over the time of tooth maturation. The feline dentin mapped in this study provides valuable insight into the spatial distribution of both essential and non-essential trace elements in feline hard dental tissues, as well as a timeline of trace element exposures.

Trace element exposure at key developmental time points can have significant health impacts later in life. In the teeth included in this study, a zoned pattern of higher uptake of barium and strontium was noted in the earliest formed coronal dentin at the level of the DEJ. This pattern is also seen in human and non-human primate teeth, and research has previously shown that barium and strontium are associated with nursing (36, 41, 42). Our findings in feline teeth suggest that nursing intake in cats also imparts clear elemental signatures in dentin in a manner similar to that of humans. Additionally, higher levels of zinc, an essential nutrient, and lead, a toxic metal, were present in the coronal dentin aligning with the nursing signal of barium and strontium. This aligns with research in humans that shows breast milk can transfer both nutrient and toxic elements from the maternal stores to the offspring (43–47). Passage of maternal stores to offspring has potential health implications, both systemic and dental. For example, lead exposure has been linked to abnormal tooth development, dental caries, and periodontal disease in human and non-human mammals (9, 48–50). Early lead exposure that changes the mineralized content of the enamel and dentin during tooth development could have structural integrity disadvantages that may predispose tooth to dental disease. Recently, studies have raised the concern of chronic trace element exposure from levels present in commercial diets for dogs and cats (18–20). This study raises the concern for transmission of lead exposure from mother to offspring, suggesting an even earlier start to chronic exposure and amplifying the need for further research in this field.

The timeline of exposure can be measured as early as prenatal development as the presence of a neonatal line (also known as birth line) in specific teeth allows LA-ICP-MS mapping of trace element exposure of that individual pre- and post-birth (4). The neonatal line is present in deciduous teeth and permanent first molar teeth as both begin to mineralize *in utero* (34). Mapping with identification of a neonatal line has been carried out in humans and non-human primates' first molar teeth to assess the potential impacts of exposure as well as nursing time lengths on systemic health (34, 41, 51). It has been shown that the first molar tooth (a primary tooth) begins mineralization *in utero* in several veterinary species, including canine, equine, and non-human primates (34, 52, 53). To the best of our knowledge, there have neither been any publications identifying the chronology of tooth development in the domestic cat nor confirming the mineralization of the first molar tooth *in utero*. While a neonatal line could not be definitively identified in the first molar teeth included in this study, the differences in nursing signature distribution between the first molar tooth (a wider band, therefore longer time) and canine tooth (a thinner band) suggest that the first molar tooth had mineralized the earliest, possibly starting *in utero*. This will become important in future studies assessing long-term exposure to trace elements in the cat, as the first molar tooth is the permanent tooth that holds the most information in an animal's life.

To date, there has been little previous research on the involvement of trace elements and dental disease in domestic cats. One previous study investigating trace elements in feline dental tissues used energy-dispersive and wavelength-dispersive X-ray microanalysis to characterize weight percentages of several trace elements in the hard dental tissue mineral content, but they did not consider toxic elements such as lead (27). Another study examined the surface mineralization differences at the CEJ of normal feline teeth, but this study only assessed the degree of mineralization rather than the composition of that mineralized dental tissue (54). To the best of our knowledge, this is the first use of LA-ICP-MS to study the microspatial distribution of multiple essential and toxic trace elements in feline teeth. This proof-of-concept study is the first to characterize trace element distributions in feline dental hard tissues, which is a needed step before applying these methods to research examining the influence of trace elements on feline dental disorders. The localization of lead in the earliest formed coronal dentin of the feline teeth included, coupled with research in other species linking lead to dental disorders, supports the need for further research to assess whether teeth affected by tooth resorption have higher lead exposure than healthy teeth over time. Examination of changes in elemental concentration adjacent to resorptive lesions was beyond the scope of this study as this would require a combination of additional analytical methods with finer temporal resolution. Additionally, the sample size of this study was small, and larger control and disease groups would be needed to compare temporal elemental distribution sufficiently. Nonetheless, the study shown here is foundational to the future study of lead and other trace element exposures in cats as well as trace element involvement in dental and systemic diseases.

## Data availability statement

The raw data supporting the conclusions of this article will be made available by the authors, without undue reservation.

## Ethics statement

Ethics review and approval were not required for the animal study because the study was not experimental and only used discarded tissues obtained from routine veterinary care. Assays were conducted on discarded teeth that had been extracted based on standard medical indications from cats, presenting for healthcare to an authorized, licensed veterinarian. This situation is not regarded as animal experimentation according to the Animal Experimentation Act. The study and its design were also discussed with the Cornell University Veterinary Clinical Studies Committee (CUVCSC), and an IACUC exemption was obtained (Protocol ID#: 030221-07).

## Author contributions

AW conceived the study, conducted the experiments, and provided the medical illustration of feline tooth in Figure 1. AW and MA wrote the manuscript. CA undertook laboratory analysis and wrote the manuscript. NF and SP supervised the sample collection and wrote the manuscript. All authors contributed to the article and approved the submitted version.
