# Supplementary material for: Microspatial distribution of trace elements in feline dental hard tissues: early life exposure to essential and toxic elements
Source: Front Vet Sci. 2023 Jun 27;10:1204210. doi: 10.3389/fvets.2023.1204210 (PMC10333753; doi:10.3389/fvets.2023.1204210)
Supplement: Supplementary file 1 [file Data_Sheet_1.docx]

**Supplemental Material**

**Microspatial distribution of trace elements in feline dental hard tissues: early life exposure to essential and toxic elements**

**Alexandra L. Wright^1*^, Nadine Fiani^1^, Santiago Peralta^1^, Manish Arora^2,3^, Christine Austin^2,3^**

^1^Department of Clinical Sciences, Cornell University, College of Veterinary Medicine, Ithaca, NY 14853

^2^Environmental Medicine and Public Health, Icahn School of Medicine at Mount Sinai, New York, NY 10029

^3^Institute for Exposomic Research, Icahn School of Medicine at Mount Sinai, New York, NY 10029

***Correspondence:**

Corresponding Author

Email: aw656@cornell.edu

**Figure S1.** **Trace elements in a diseased first molar tooth.** A mandibular first molar tooth extracted from a cat due to tooth resorption lesions. Linear distributions for barium (A), strontium (B), lead (C) and zinc (D) are shown from the level of the dentin horn to cementoenamel junction (CEJ). Feline ID: 119-309. Higher levels of elements are noted in coronal dentin, which then taper off when sampled near the CEJ.

**Figure S2.** **Trace elements in a diseased first molar tooth.** A mandibular first molar tooth extracted from a cat due to tooth resorption lesions. Linear distributions for barium (A), strontium (B), lead (C) and zinc (D) are shown. Feline ID: 189-309. A banding pattern of alternating high and low intensity is noted in all four elements, and the pattern of tapered intensity when closer to the CEJ is present.
